# Address to the Association of Veterinary Faculties of North America1Read at Des Moines, Iowa, September 10, 1895.

**Published:** 1896-01

**Authors:** J. H. Wattles

**Affiliations:** The Kansas City Veterinary College


### ADDRESS TO THE ASSOCIATION OF VETERINARY FACULTIES OF NORTH AMERICA.[1]

BY J. H. WATTLES, V.S.,
OF THE KANSAS CITY VETERINARY COLLEGE.

WHILE the subject of this address is ostensibly that of the results that have been obtained by prescribed entrance examinations, and as the subject is to-day largely prospective as far as my individual experience goes, my fellow-members will pardon me if some digression is made, and some of the results considered that may be obtained by having positive and fixed lines for entrance examinations to be conducted upon, as well as some of the conditions that exist and evils that might result were each school allowed to act as its own censor in the matter of matriculation and graduations.

With the organization of the several faculties of the veterinary medical colleges of the country in the Association of Veterinary Faculties of North America the fraternity and public had every reason to believe that a new era had dawned upon our chosen profession, and that, while the pathway might not be strewn with roses, the way was clear for our kindred professions to see that we were entitled to stand in the front rank of the learned professions and that we were capable of maintaining that rank.

With solely fraternal and educational objects in view, the Association of Faculties was formally organized in Philadelphia last September, and, to those who had the pleasure of attending that meeting, will always remain a remembrance of the kindly spirit in which all of the deliberations were maintained. Without acrimony or intemperate language, and with a full and

[1] Read at Des Moines, Iowa, September 10, 1895.

generous consideration of the many different conditions and surroundings of the several schools, the constitution of the Association was adopted, and I earnestly believe with liberal ideas on the subject of advanced education we meet again to-day.

There is no reasonable doubt existing in the mind of any impartial and disinterested person of the necessity of a higher standard of educational requirement in the matriculation examinations of our prospective students, and we can all see very plainly that it is only a matter of a very short time when every college that will be recognized as coming up to the standard will have adopted a minimum grade for the admission of students. With these points so clearly in view, and with the upward and onward tendency of the leaders in our profession, and with positive knowledge that different States are formulating laws regulating veterinary practice, it seems to me to be a rational conclusion that the proper thing for us to do is to act in the matter at once, and not wait until compelled to act, and for all of the colleges of this continent to unite with this association in endeavoring to create a certain standard in the admission of students, in their advancement in their respective studies, the length of time they shall be required to attend, and in their final graduation.

By having positive rules laid down for the admission of students every one will be perfectly satisfied that the gentlemen who graduate are capable, educated men. We will then be assured that men are not allowed to graduate simply because they have been in empirical practice for a time, or because they are druggists, or because they hold some classical degree or a diploma from some normal school. On the contrary, we will know that they are men that have been educated for the veterinary profession, in veterinary colleges, and are capable of practising in that profession.

It is important to us all that the moral character and business principles of men about to enter our profession be inquired into before they are allowed to matriculate. My belief is that every applicant should be required to fill out a printed blank agreeing to comply with all of the rules of the college he wishes to enter, and also agreeing to comply with the code of ethics of the State in which he may enter practice. This surely would be no hardship for any honorable man, and we do not desire any other in the profession. It is surely a very embarrassing thing for us to come in contact in our practice with men that are graduates of recognized colleges and to feel that we do not care to affiliate with them because of some underhand business methods, or because they are drunkards or gamblers, and it seems to me that close scrutiny should be made of intended students before admitting them.

One very important matter that needs the close attention of this Association is that of the actual time of attendance of students. It seems to me to be a ridiculous proposition to say in an announcement that we require attendance on a certain number of courses of six months each, and then allow men to attend only a fraction of the time. To illustrate this phase of the subject: Suppose a student enters in November, 1893, and he is allowed to come up for final examination the following spring, and is passed, the student's name appearing in the list of graduates in the catalogue issued in 1894. Could this student have attended more than four and a half months actual time?

Suppose the fact to exist that schools were in the habit of allowing men to enter the school and appear for final examination at the end of one school year, and even the year shortened by the late appearance of the student. Could a student acquire sufficient knowledge in so limited a time to become proficient in our science? The men usually applying for time allowance are the practitioners from some small town where there had been no qualified veterinarian until a man recently graduated entered the neighborhood and forced the older practitioner to protect himself by gaining a diploma, or they are forced into college by the action of State laws. My experience with this class of men, as students, is that they are the slowest to grasp ideas in school, and make the poorest practitioners when out.

The requirements of the college that I have the honor to represent, during the first two years of its existence, were the same as in other two-year colleges, and I can look back over our brief period of existence and see nothing to regret, except the fact that we admitted during our first two years men that had been in practice for some time, and allowed them to appear for final examination at the end of the first year. Happily for us, we were soon satisfied with the experiment, and the practice was discontinued. During the same period we allowed licentiates and graduates in pharmacy to enter the senior class, but we soon discovered that the time was too limited for them to acquire sufficient knowledge, and this practice was also abolished.

For the past two years our matriculation examination has been up to the standard required by this Association, consequently we were in no manner disturbed by adopting the rules of this body, and, unless my judgment is very much at fault, there is not a college in America that would not be benefited by adopting the same rules and maintaining a rigid adherence to them.

All applicants for matriculation in this college are now required to fill out the printed application furnished by the school, in which they agree to conform to the established rules. All applications for admission are acted upon at a regular meeting of the faculty, and the application must be approved by three-fourths of the number present at such meeting. This takes the responsibility out of the hands of one man, and places it where it justly belongs, in the hands of the entire faculty. We require an actual attendance at 80 per cent. of all lectures, and no student is allowed to come up for examination that has not attended the required percentage. This does away with the practice of students entering and leaving school at will, or inattendance upon lectures or other forms of instruction.

Every inducement is held out to our students to take the three years' course, with the result that 50 per cent. of those who entered last year have accepted, and fully as large a percentage will begin the three years' course this session. As we have not felt strong enough as yet to put on a compulsory three years' course, we have met the condition as far as we possibly could by making an optional course of three years, from which is but a short step to the regular course prescribed by the representative body of our profession, and now adopted by the leading colleges of America.

With the firm conviction that in union there is strength, and with every desire to advance the interests of our profession, with the belief that it is only by union in action, kindness in our discussion, charity to all, that we can reach the goal of our ambition in our life-work, I thank you for the honor you have conferred upon me by giving me your attention.

[Since preparing this address for this Association, the Kansas City Veterinary College has decided upon a three-year compulsory course, beginning January 1, 1896. This present session is the last that students will be admitted for a two-year course.—J. H. W.]

